# Can Physical Activity, Sleep Parameters, and Sleep–Wake Patterns Predict Outcome of Combined Chronotherapy in Mood Disorder During Routine Clinical Practice? An Exploratory Study

**DOI:** 10.3390/jpm16020100

**Published:** 2026-02-07

**Authors:** Stella J. M. Druiven, Olga Minaeva, Benno C. M. Haarman, Ybe Meesters, Robert A. Schoevers, Jeanine Kamphuis, Harriëtte Riese

**Affiliations:** Department of Psychiatry, University Medical Center Groningen, University of Groningen, 9700 RB Groningen, The Netherlands

**Keywords:** combined chronotherapy, sleep deprivation, light therapy, actigraphy, depression

## Abstract

**Background/Objectives**: Combined chronotherapy (CCT), which combines repeated sleep deprivation and light therapy, is used in the clinical treatment of severe depression. Despite its potential to rapidly reduce depressive symptoms, CCT is infrequently used in clinical practice. We explored whether actigraphy-derived within-patient changes in physical activity, sleep parameters, and sleep–wake patterns prior to CCT can help identify those most likely to benefit from this treatment, supporting personalized mental health care. **Methods**: Actigraphy data from nine severely depressed patients were collected before, during, and after CCT. Data were assessed with a questionnaire on depressive symptoms (Inventory of Depressive Symptomatology—Self Report, IDS-SR) and actigraphy measures for sleep–wake patterns and physical activity: daily mean activity level, rhythm (intradaily variability (IV), interdaily stability (IS)), Midpoint of Sleep (MSF), time in bed, sleep efficiency (SE), and the fragmentation index (FI). Variables were compared before and after CCT by systematic visual inspection due to the small sample size. A prior set Minimal Clinically Important Difference (MCID) of a 30% change in IDS scores from before and the week after CCT was used to categorize patients as responders (n = 3) or nonresponders (n = 6) to CCT. **Results**: After CCT, for both responders and nonresponders, there was a notable decrease in IDS, IV and FI. Prior to CCT, responders, compared to nonresponders, were characterized with higher IDS, more time in bed and higher FI, while having lower SE. **Conclusions:** We concluded that actigraphy assessments during regular CCT are feasible and found preliminary evidence that patients with the most disrupted sleep–wake patterns prior to treatment may benefit most from CCT.

## 1. Introduction

Alterations in circadian rhythms, clinically reflected in diurnal mood variations and sleep difficulties, are common in mood disorders [1]. These disruptions are particularly pronounced in Major Depressive Disorder (MDD) and Bipolar Disorder (BP), where they are considered a core pathophysiological feature of the disorder [2]. A well-established, intensive therapy that specifically targets resetting the circadian rhythm is combined chronotherapy (CCT). The combination of repeated sleep deprivation (SD) and light therapy (LT) is a type of CCT used to treat severe depressive symptoms with the intention of improving mood and stabilizing sleep and sleep–wake patterns [1]. Interest in CCT started with the discovery of the rapid positive effect on mood after a single night of SD, which diminishes following a night of recovery sleep [3]. To prolong the beneficial effects on mood, SD has been combined with other forms of CCT such as LT [4].

Currently, CCT is used in the clinic to treat patients suffering from a depressive episode in both bipolar and unipolar depression [5,6]. Despite the known potential, its use in the clinic is limited [7]. This is likely partially due to the limited information available regarding which patients will benefit from CCT, a gap that if addressed, could inform personalized treatment decisions for individual patients. Some evidence for markers that possibly predict a positive response to SD alone and combined with LT is reported for a patient’s self-reported chronotype (evening-type) and positive diurnal mood variation (mood best in the evening) [8]. Furthermore, a higher level of observed physical activity on the day before SD predicts a positive response to SD [9]. Identifying ways to measure chronotype, diurnal mood variation, and physical activity in a clinical setting may help predict CCT response more precisely in individual patients. Actigraphy provides an unobtrusive way to measure physical activity, sleep parameters, and sleep–wake patterns [10]. Time series data assessed with actigraphy are used to calculate non-parametric markers of sleep–wake pattern stability [11]. As CCT is used to stabilize patient’s sleep and sleep–wake patterns, it is expected that these markers change after CCT. Supporting this hypothesis, in their systematic review, Panchal and colleagues (2022) showed that therapies specifically targeted at the circadian rhythm (e.g., light therapy, cognitive behavioral therapy for insomnia, blue blocking glasses) stabilized sleep and sleep–wake patterns as indexed with actigraphy-derived markers in patients diagnosed with a Bipolar Disorder [12].

Previous research established the efficacy and feasibility of CCT [4,8,12]. A study that included actigraphy assessments at baseline before CCT for patients with a unipolar depression assessed sleep characteristics and did not analyze whether these could predict a positive response to CCT [13]. These studies have not yet incorporated actigraphy to assess potential predictive markers of individual patients’ responses to CCT. Moreover, like most medical studies, these studies used a group-based (nomothetic) approach to contribute to knowledge about generalized patterns across the sample. However, treatment in daily clinical practice demands insights into the psychopathological characteristics of a specific individual patient. To enrich personalized medicine, research approaches that take into account between-patient heterogeneity, as well as within-patient longitudinal changes during treatment and symptom fluctuations, are urgently needed [14,15]. A well-established research approach to do so is the individual-based, single-case design approach, which has wide applicability in health-related research and practice [16,17]. In this approach, the value of a study lies not primarily in the number of subjects, but in the number of repeated observations collected per subject.

In this exploratory clinical study, actigraphy and questionnaires were used to monitor a small number of patients treated with CCT for their depressive symptoms in regular clinical practice. The study had the following aims: (1) examine whether actigraphy can be used to obtain markers on physical activity, sleep, and sleep–wake patterns during CCT; (2) compare actigraphy-derived markers before and after CCT; (3) explore whether actigraphy could be used to search for predictors of response to CCT in future studies.

## 2. Materials and Methods

### 2.1. Patients

The inclusion criteria for referral to CCT were a diagnosis of a depressive episode (either due to an MDD or BP [18]) based on DSM-V criteria and a minimum age of 18 years. Exclusion criteria included experiencing manic symptoms, psychotic symptoms or epilepsy. After referral by their doctor, patients were seen in the clinic for an intake interview with a psychiatrist before the start of CCT. The recruitment period lasted from 1 December 2018 until 1 March 2022, which included the COVID-19 pandemic period, hampering inclusion. Before inclusion, patients were fully informed and provided written informed consent.

### 2.2. Combined Chronotherapy

Data were collected during care-as-usual with CCT in the University Center for Psychiatry at the University Medical Center Groningen (UMCG), a specialized tertiary center in the Netherlands.

Patients were admitted to the clinic in the week before the start of CCT and stayed as inpatients during the weeks of SD and LT, a total of 2–3 weeks. Most patients went on leave during the weekends and returned before the start of an SD night on Sunday.

**Figure 1:**
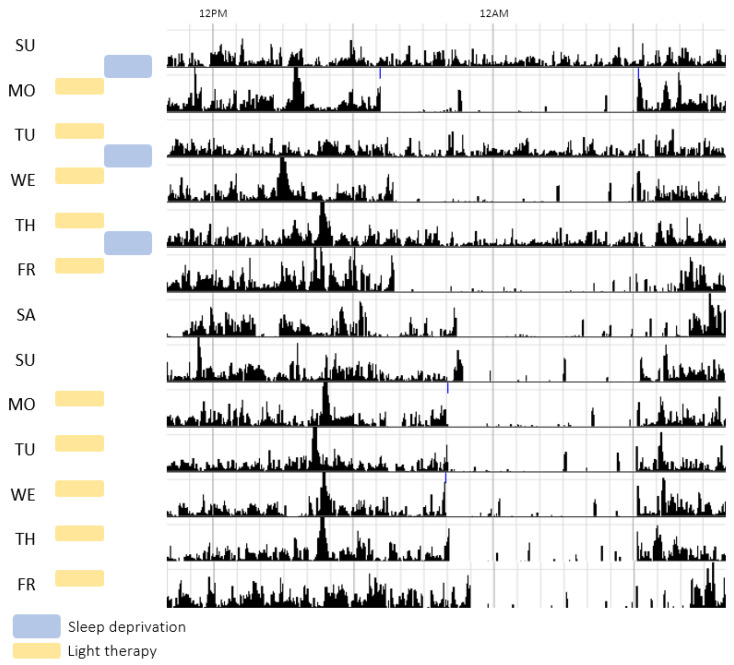
Example of actigraphy data of a single patient during the two weeks of combined chronotherapy. The level of activity (counts) is depicted as black bars across the day; each row represents one day. Timing of sleep deprivation nights (blue bars) and light therapy periods (yellow bars) are indicated by the bars on the left side.

As depicted in Figure 1, the first week of CCT included three nights of SD on Sunday, Tuesday, and Thursday with recovery nights in between. Patients were allowed to sleep from 6:00 PM on the day following SD until 7:00 AM the next day. During the nights of SD, patients were accompanied by a CCT protocol-trained night nurse and were encouraged to stay awake by doing activities such as games and walks. Light therapy was administered every weekday from 7:00 to 7:30 AM for two weeks, starting the morning after the first SD night (Monday). A light intensity of 10,000 LUX was used for 30 min (SunSquare, The SunBox® Company, Gaithersburg, MD, USA). The protocol was evaluated by the Medical Ethics Review Board of the UMCG as not falling under the scope of the medical research involving Human Subjects Act (WMO; M18.233069). In addition, since the study does not involve a clinical trial as defined by applicable regulations, clinical trial registration was not required, although the study was registered in the UMCG Research Register (RR-number 201800513). Data collection and analysis complied with all relevant ethical guidelines, including those set out by the Medical Ethics Review Board of the UMCG.

### 2.3. Questionnaires

Information on sex, age, and clinical diagnosis was collected from the patient’s electronic health record. Assessment of severity of depressive symptoms was done weekly with the Inventory of Depressive Symptomatology—Self Report (IDS-SR) [19]. Response was calculated at the end of the final week of CCT or, for the patients with a missing assessment in the final week, the week after CCT. For determining response/nonresponse, the Minimal Clinically Important Difference (MCID) was used [20]. For the IDS-SR, no MCID has been determined yet. Based on the MCID scores of other depressive symptom scales, a decrease of ≥30% in IDS-SR score was considered a significant decrease in depressive symptoms and was labeled as ‘response’ [20]. For three patients, two IDS-SR assessments were available after CCT; for them, response was calculated with the first assessment. The exact timing of IDS-SR assessments are given in Figure S1.

### 2.4. Actigraphy Assessments and Pre-Processing

For the actigraphy assessment, the MotionWatch8 (MW8) was used (CamNtech; Cambridge, UK). The device sampled tri-axis acceleration at 3–11 Hz and generated activity counts (ACs) with an epoch length of 60 s. Assessments started as soon as the therapy was planned, which often meant that the MotionWatch8 was sent to the patient’s home by regular mail. Patients were asked to wear the watch on the non-dominant wrist and to only take it off when going to the sauna. The number of days of actigraphy monitoring differed for each patient due to the timing of the intake interview and the actual start of chronotherapy but included at least 6 days before CT, 14 days during CT, and at least 6 days after CT. Patients were instructed to press the event marker button when they were going to sleep (i.e., not going to bed) and when they woke up (i.e., not when getting out of bed). Besides wearing the MotionWatch8, they were asked to fill in a sleep diary with the times of going to bed and waking up.

Sleep–wake parameters were computed using the ACTman software package (2017) [21] by applying the NPCRA (non-parametric circadian rhythm analysis) method [11]. The NPCRA includes calculating IS (interdaily stability, or stability of rhythm from one day to the next) and IV (intradaily variability, or stability of rhythm within a day, the rate of shifting from rest to activity). IV was calculated per day, while IS required multiple days of observations and was calculated on two consecutive days of data. The native MotionWare software (version 1.3.17) was used to calculate the following daily sleep variables: time in bed (difference between bedtime and rise time (hours)), sleep efficiency (SE: percentage of time asleep from total time in bed), and fragmentation index (FI: percentage depicting restlessness during sleep). Additionally, we calculated the midsleep on work-free days (MSF), which is the midpoint between sleep onset and offset on free days, and daily mean activity [22].

### 2.5. Analyses

To explore within-patient individual changes from baseline to after CCT, delta-scores of the IDS-SR and actigraphy data were calculated (Δ score = ‘after CCT’−baseline’) and plotted separately for each patient. Given the small sample size, systematic visualization of individual data rather than relying on group-level statistical comparisons offers a more transparent representation of variability and potential trends [23]. To further examine whether responders to CCT could be distinguished at baseline, all baseline parameters were plotted separately for MCID-defined responders and nonresponders.

## 3. Results

### 3.1. Patients

Sociodemographic and clinical descriptives are given in Table 1. There was an equal distribution of diagnoses: seasonal MDD (n = 2), non-seasonal MDD (n = 3), BP type 1 (n = 3), and BP type 2 (n = 1). All patients had previously received multiple types of pharmacological anti-depressant treatments and most (n = 8) had been previously admitted to a psychiatric hospital or treated during day-treatment for their depression. Three patients were categorized as responders in the final week or week after CCT, and six were categorized as nonresponders.

### 3.2. Individual Change in Markers After CCT

**Figure 2:**
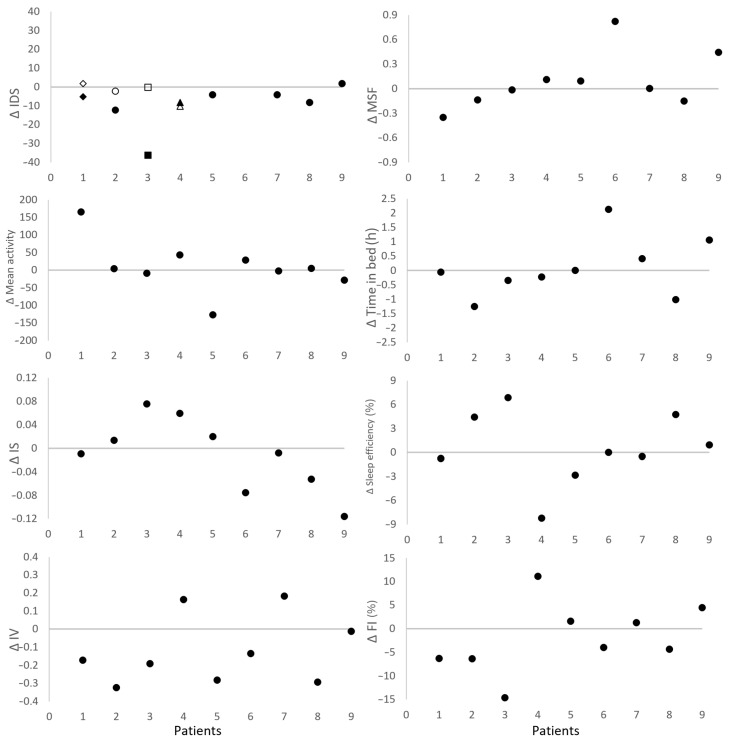
Change in physical activity, sleep and sleep–wake patterns after combined chronotherapy (CCT) (Δ score = ‘after CCT’—‘baseline’ on the y-axis) per patient (n = 9, on the x-axis), where each patient is depicted as one dot in the graph. As described in the method section, response was defined as a ≥30% decrease in IDS-SR from baseline to the last week of CCT (see method section for details). Patient #3 had two IDS-SR measurements before CCT; Δ scores calculated using the first (□) and second (■) score are shown. Patients #1, #2 and #4 had two IDS-SR measurements after CCT; Δ scores calculated with the first (open symbol (white circle)) and second (closed symbol (black circle)) score are shown. IDS: inventory of depressive symptoms—self-report; MSF: midsleep on free days; mean activity: daily mean activity; IS: interdaily stability; FI: fragmentation index; IV: intradaily variability.

An overview of all parameters before, during, and after CCT for each patient is shown in Figure S1. In Figure 2, the change in markers after CCT is shown. Visual inspection revealed that most patients showed a decrease in IDS-SR, IV, and FI after CCT.

### 3.3. Baseline Markers in CCT Responders and Nonresponders

**Figure 3:**
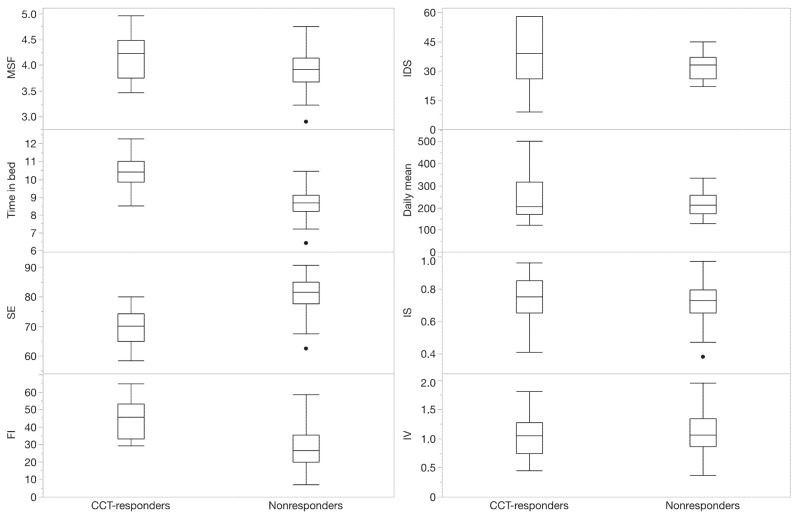
Baseline severity of depressive symptoms, physical activity, sleep parameters, and sleep–wake patterns in combined chronotherapy (CCT) responders and nonresponders (for details on categorization, see method section). MSF: midsleep on free days; SE: sleep efficiency; FI: fragmentation index; IDS: inventory of depressive symptoms—self-report; IS: interdaily stability; IV: intradaily variability. The boxplot shows the median as the center line, with the 25th and 75th percentiles as the top and bottom of the box. The whiskers represent the expected variation of the data, with outliers shown as dots.

Figure 3 shows the difference in baseline parameters for the CCT responders versus nonresponders. Compared to nonresponders, responders showed higher IDS-SR scores, longer time in bed and FI values, and a lower SE.

## 4. Discussion

In this exploratory study, we demonstrated that actigraphy is an easy-to-use tool for obtaining markers of physical activity, sleep parameters, and sleep–wake patterns in patients undergoing CCT in regular clinical practice. The results indicated that baseline actigraphy data may provide useful information systematically related to CCT response, as patients who responded positively to CCT had a higher baseline FI and spent more time in bed, while exhibiting lower SE. We tentatively conclude that it may be valuable to investigate these markers as predictors of response in individual patients in a larger study. These findings could contribute to the development of personalized treatment strategies for depression.

A notable advantage of using actigraphy for assessing physical activity, sleep and sleep–wake patterns is the amount of objective information that can be passively collected during routine care. This adds to routine clinical data collection with self-report questionnaires on mood and sleep measures, as often this subjective information does not strongly correlate with objective measurements [25]. Another advantage, although not formally assessed, is that patients reported no side effects or made other negative comments related to wearing the actigraphs. Actigraphy is increasingly utilized in research to assess physical activity, sleep parameters, and sleep–wake patterns in mood disorders, underscoring the importance of exploring its potential for other applications, such as identifying subgroups for clinical staging [26] or, as suggested in the current paper, predicting individual treatment response.

In the group of patients receiving CCT, we found an overall decrease in depressive symptoms, FI, and IV after CCT treatment. The 33% response rate in this sample was lower compared to earlier reports of 40–60% [5]. Yet our sample was small, and since data were collected in routine care, not all data were complete. The percentage of response was similar in the study of Sikkens et al. (2019) performed in the same hospital, who reported a response rate of 34% to CCT including 26 depressed patients [6]. Moreover, there is a relatively high number of patients with treatment-resistant depression that are treated at our tertiary academic psychiatric center. The patients of the current study had all previously received at least two types of antidepressant medication. To compare, in the Sequenced Treatment Alternatives to Relieve Depression (STAR*D) trial, the remission rate at levels 3 and 4 (third and fourth switch of antidepressant medication because of nonresponse) was 14% and 13% [27]. A previous study by Benedetti et al. also reported a lower response rate (50% reduction in depressive symptoms on the last day of combined CCT) for medication-resistant patients (44%) versus non-resistant patients (70%) [28]. Two out of three of our responders in the final week of CCT were no longer responders in the week after CCT. This reflects the difficulty in maintaining CCT’s effect after the initial response, which has been reported before. A significant decrease in depressive symptoms one week, but not two weeks after treatment with SD was reported in a meta-analysis on the efficacy of SD, combined with standard care in the treatment of depression [29]. Adding other chronotherapeutic approaches may be helpful to maintain the effect for a longer period, such as sleep phase advance or social rhythm therapy [5,6].

Our study had multiple limitations. First, the study had a small sample size with a large age range and there was no control group. Because of that, the data collected could not be tested by statistical analyses and results could not be compared between men and women, making the results tentative and potentially limiting their replicability in larger samples. Additionally, the sample consisted of mixed diagnoses (MDD, BP), resulting in a less homogeneous sample, which may further limit the interpretability and generalizability of the results. To ensure transparency, we combined the visual inspection of the data in the results section with complete patient-level results provided in the Supplementary Materials (Figure S1). Nevertheless, replication in a larger sample is necessary, together with the use of robust research designs that capture both between-patient heterogeneity and within-patient longitudinal changes during treatment, as well as symptom fluctuations [30]. Second, in addition to the small sample size, data were not complete for every patient, which could have introduced a potential bias in the interpretation of the results. For example, because of the missing data, response to CCT was calculated using either the timepoint at the end of the CCT or the subsequent week. The percentage of responders may have been different if the response was calculated using the same timepoints for every patient. The main reason for the missing data is that the study was performed during regular care and not as part of a clinical trial. However, this real-world setting provides valuable insights into the practical feasibility of incorporating innovative technologies like actigraphy into routine clinical practice. Moreover, an MCID for the IDS-SR has not been established yet. As explained in the Method section, based on the MCID scores of other depressive symptom scales, a decrease of ≥30% in IDS-SR score was considered a significant decrease in depressive symptoms in this study [20]. Using another percentage might have shifted the categorization of the patients into the responder/nonresponder groups and the subsequent visual trends described in the results because of the small sample size. Third, most patients were using medication during the CCT period (e.g., benzodiazepines, antidepressants, mood stabilizers; see Table 1), which may have influenced physical activity, sleep, and sleep–wake patterns [31]. Because this study was conducted in a routine clinical setting, these effects could not be controlled for, but they should be considered when interpreting the findings. A notable strength of our study is that, to our knowledge, we were the first to explore physical activity, sleep parameters, and sleep–wake patterns on an individual patient level with actigraphy in CCT in a routine clinical setting. Systematic information assessments at the individual level can enrich and guide personalized treatment strategies, individualized predictions, and shared decision-making [32]. Nevertheless, both group-based and single-case design approaches have their limitations and strengths [14]. Ideally, they should complement each other to bridge the research and clinical practice gap, facilitating personalized medicine.

## 5. Conclusions

From this exploratory study, we tentatively conclude that actigraphy may be a useful add-on tool to CCT to assess physical activity, sleep, and sleep–wake patterns during regular clinical practice. In line with previous research, CCT was found to stabilize depressed mood, sleep, and sleep–wake patterns. Furthermore, patients with more disrupted sleep–wake patterns prior to treatment may benefit most from CCT. In the future, actigraphy may hold the potential to predict individual patient responses to CCT. This could support the development of more personalized treatment strategies for depression.

## Figures and Tables

**Table 1:** Sociodemographic and clinical descriptives.

Characteristics	Median	Range	n (%)
**Sex**			
Women			4 (44)
Men			5 (56)
Age (years)	58	38–69	
**Education level** *			
Low			1 (13)
Mid			4 (50)
High			3 (38)
**Diagnosis**			
MDD			5 (56)
Seasonal			2
Non-seasonal			3
BPI			3 (33)
BPII			1 (11)
**IDS**			
Baseline	37	9–58	
Final	27	11–37	
# of previous pharmacological antidepressant treatments	4	2–10	
# of previous admissions in psych. hospital or day-treatment programs	1	0–3	
**Psychiatric medication (at admission)**			
SSRI			1
SNRI			4
TCA			2
Mood stabilizers			9
Benzodiazepines			7
Antipsychotics			2

* The Dutch institute: Standard Classification of Education (Standaard Onderwijsindeling (SOI)) of the Office of Statistics (Centraal Bureau voor de Statistiek (CBS)) was used to score level of education [24]; information on education level of one participant is missing. MDD: Major Depressive Disorder; BPI: Bipolar Disorder Type 1; BPII: Bipolar Disorder Type 2; IDS: Inventory of Depressive Symptomatology; SSRI: Selective Serotonin Reuptake Inhibitor; SNRI: Serotonin and Norepinephrine Reuptake Inhibitor; TCA: Tricyclic Antidepressant.

## Data Availability

The data presented in this study are available on request from the corresponding author due to privacy restrictions.

## Supplementary Materials

The following supporting information can be downloaded at: https://www.mdpi.com/article/10.3390/jpm16020100/s1, Figure S1: Figures of depressive symptoms, physical activity and sleep–wake patterns for each patient before, during and after chronotherapy.

## Abbreviations

The following abbreviations are used in this manuscript:

CCT	Combined Chronotherapy
SD	Sleep Deprivation
LT	Light Therapy
IV	Intradaily Variability
IS	Interdaily Stability
MSF	Midpoint of Sleep on Free Days
SE	Sleep Efficiency
FI	Fragmentation Index
MCID	Minimal Clinically Important Difference
MDD	Major Depressive Disorder
BPI/II	Bipolar Disorder Type I/Type II
IDS-SR	Inventory of Depressive Symptomatology—Self Report
MW8	Motion Watch 8
AC	Activity Count
UMCG	University Medical Center Groningen
STAR*D	Sequenced Treatment Alternatives to Relieve Depression
