# Supplementary material for: Can Physical Activity, Sleep Parameters, and Sleep–Wake Patterns Predict Outcome of Combined Chronotherapy in Mood Disorder During Routine Clinical Practice? An Exploratory Study
Source: J Pers Med. 2026 Feb 7;16(2):100. doi: 10.3390/jpm16020100 (PMC12941795; doi:10.3390/jpm16020100)
Supplement: Supplementary file 1 [file jpm-16-00100-s001.zip › jpm-4056651-supplementary.pdf]

Supplemental materials to: Can physical activity, sleep parameters, and sleep-wake patterns predict outcome of combined chronotherapy in mood disorder during routine clinical practice? An exploratory study

S.J.M. Druiven, O. Minaeva, B.C.M. Haarman, Y. Meesters, R.A. Schoevers, J. Kamphuis, H. Riese

University of Groningen, University Medical Center Groningen, Department of Psychiatry, Groningen, The Netherlands

Figures S1 – Figures of depressive symptoms, physical activity and sleep–wake patterns for each patient before, during and after chronotherapy.

Note: The axes of the variables differ in range per patient, with the intention of increasing the readability of the figure. IDS: inventory of depressive symptoms – self report; Daily mean: daily mean activity; IS: interdaily stability; IV: intradaily variabilty; MSF: midsleep on free days; time in bed (hours); SE: sleep efficiency (%); FI: fragmentation index.

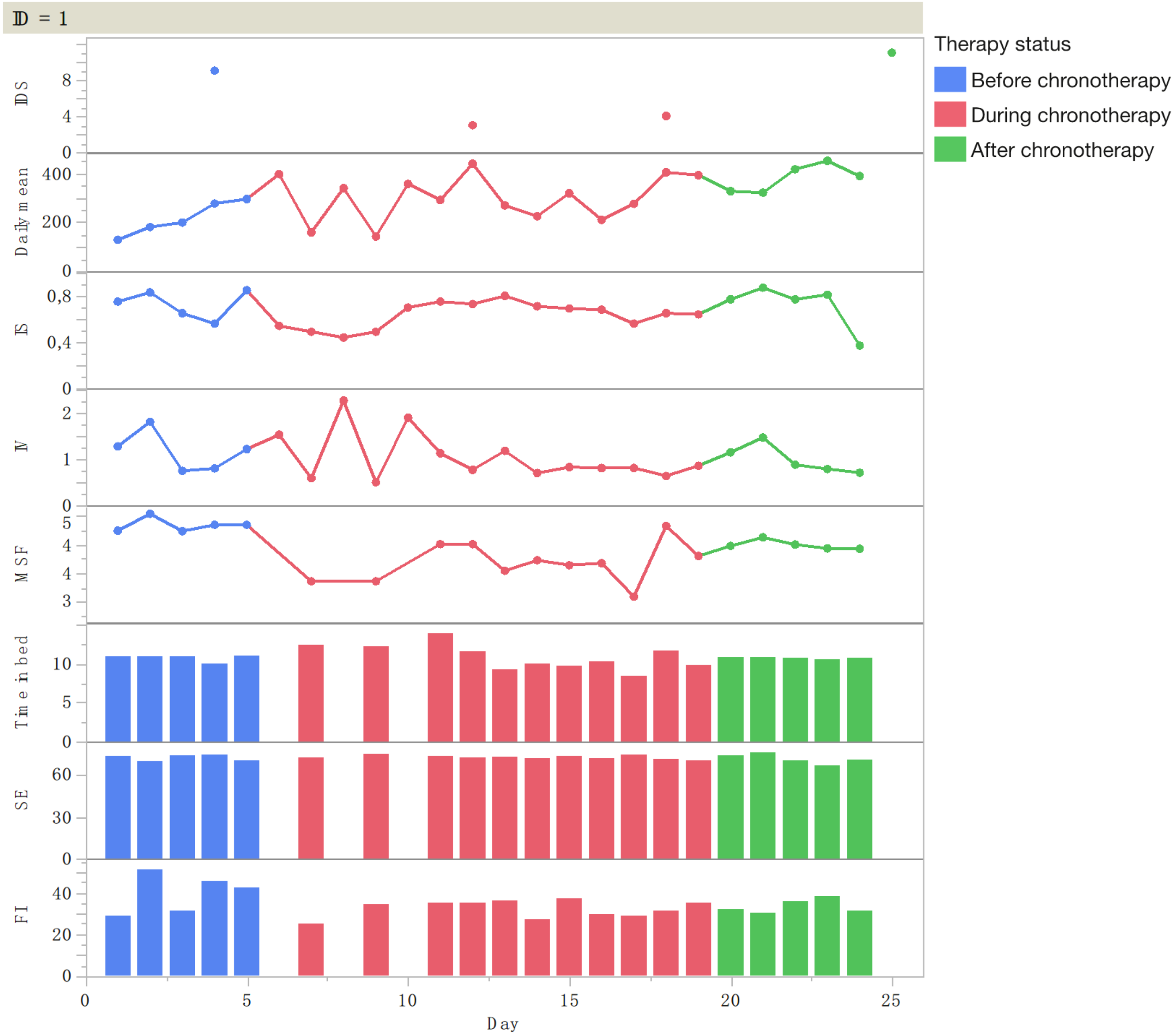

Note: For the IDS, the third assessment on day 19 at the end of chronotherapy was taken as an 'after chronotherapy' measure for the calculation of response/nonresponse.

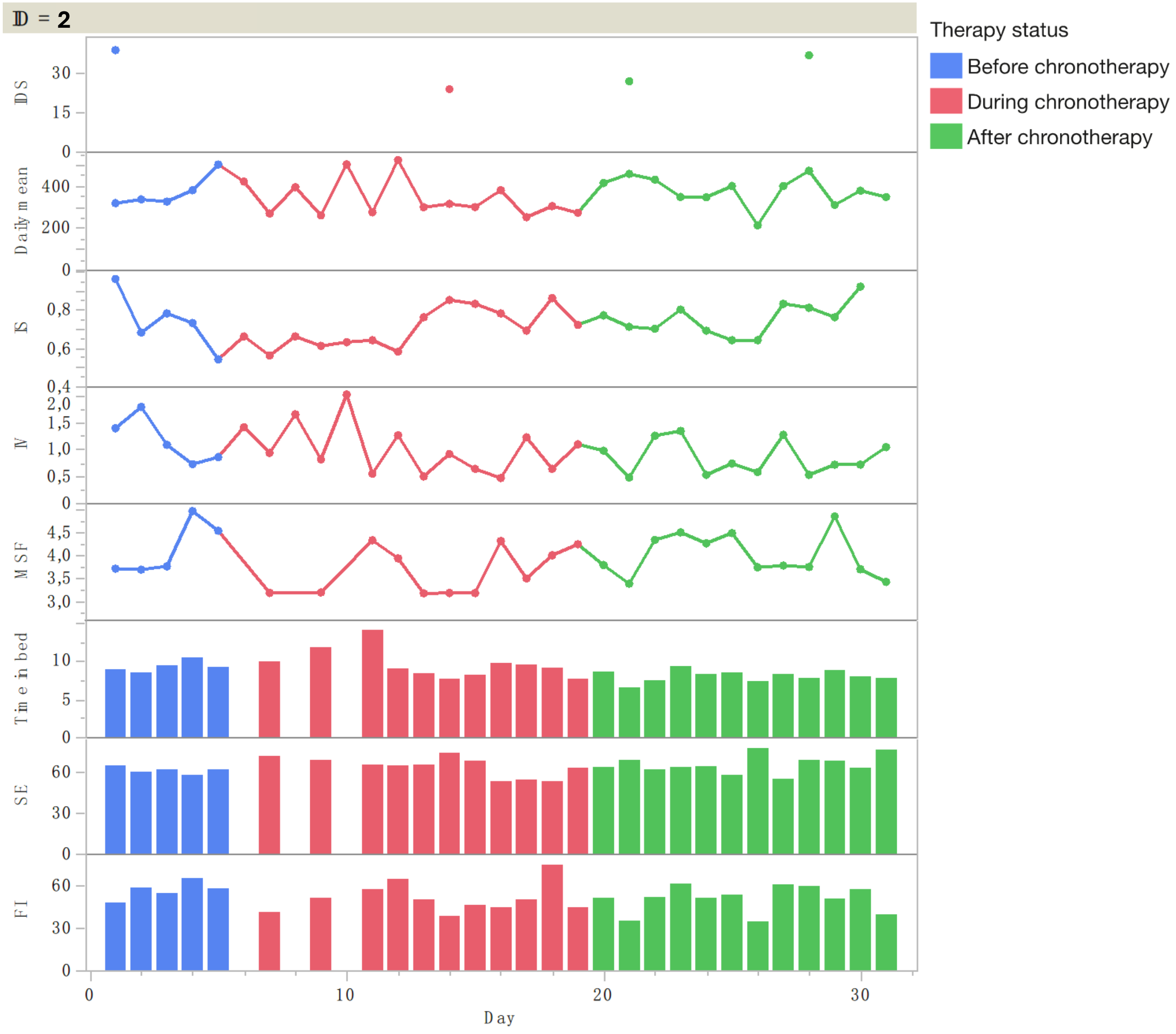
ID = 2
Therapy status
Before chronotherapy
During chronotherapy
After chronotherapy
IDS
Daily mean
IS
IV
MSF
Time in bed
SE
FI
Day

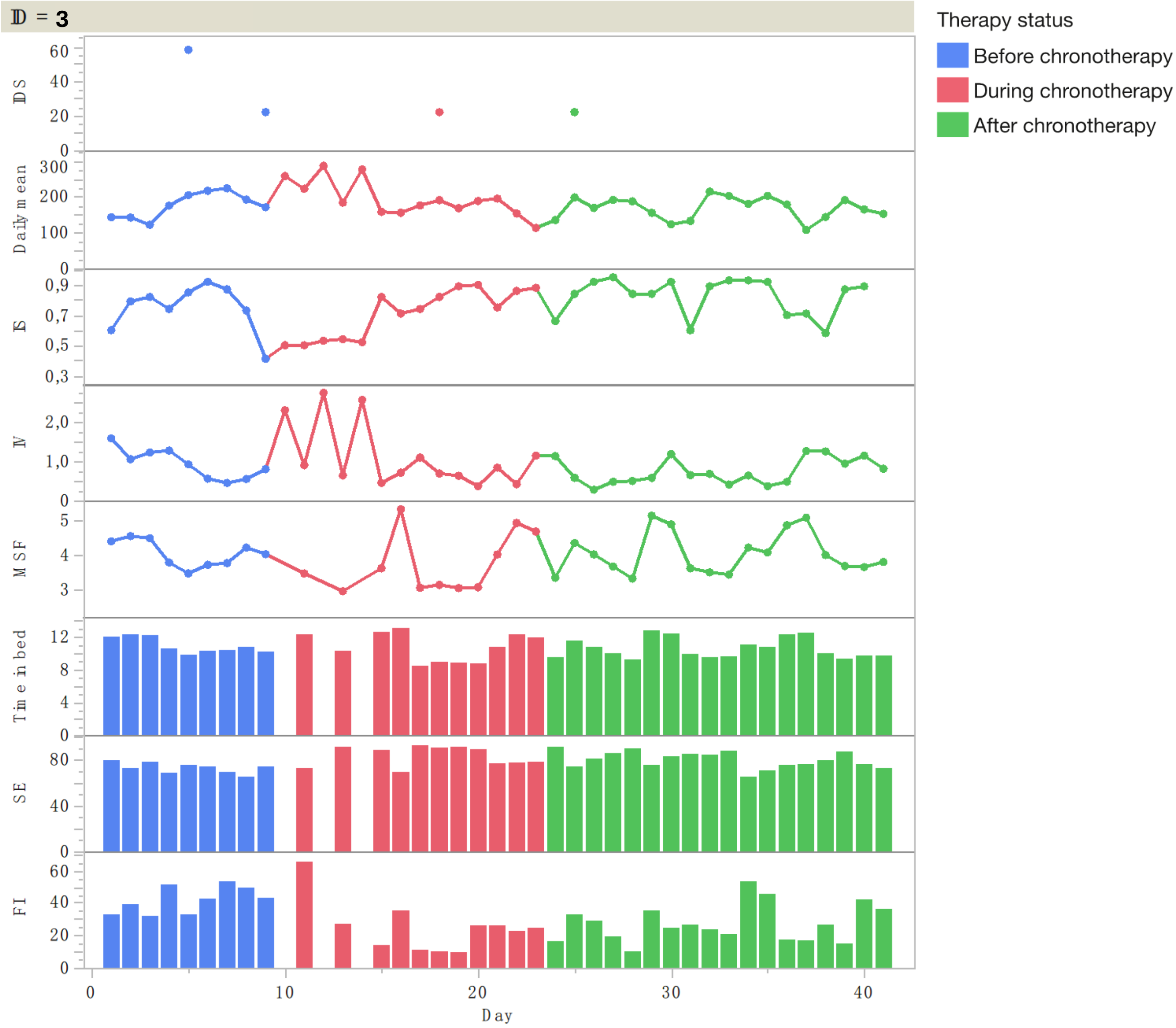

ID = 3
IDS
Daily mean
IS
IV
MSF
Time in bed
SE
FI
Day
Therapy status
Before chronotherapy
During chronotherapy
After chronotherapy

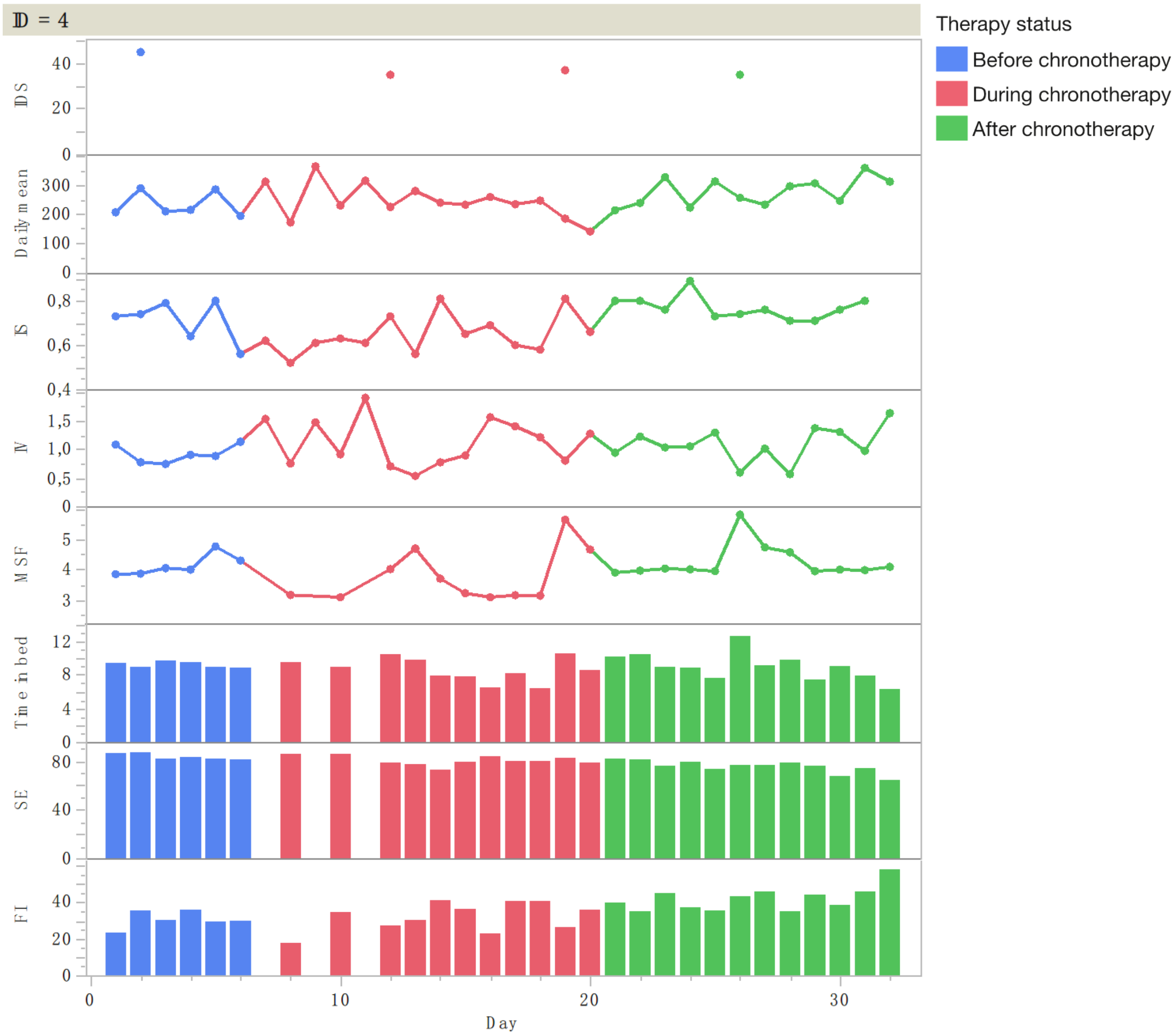

Note: For the IDS, the third assessment on day 19 at the end of chronotherapy was taken as an ‘after chronotherapy’ measure for the calculation of response/nonresponse.

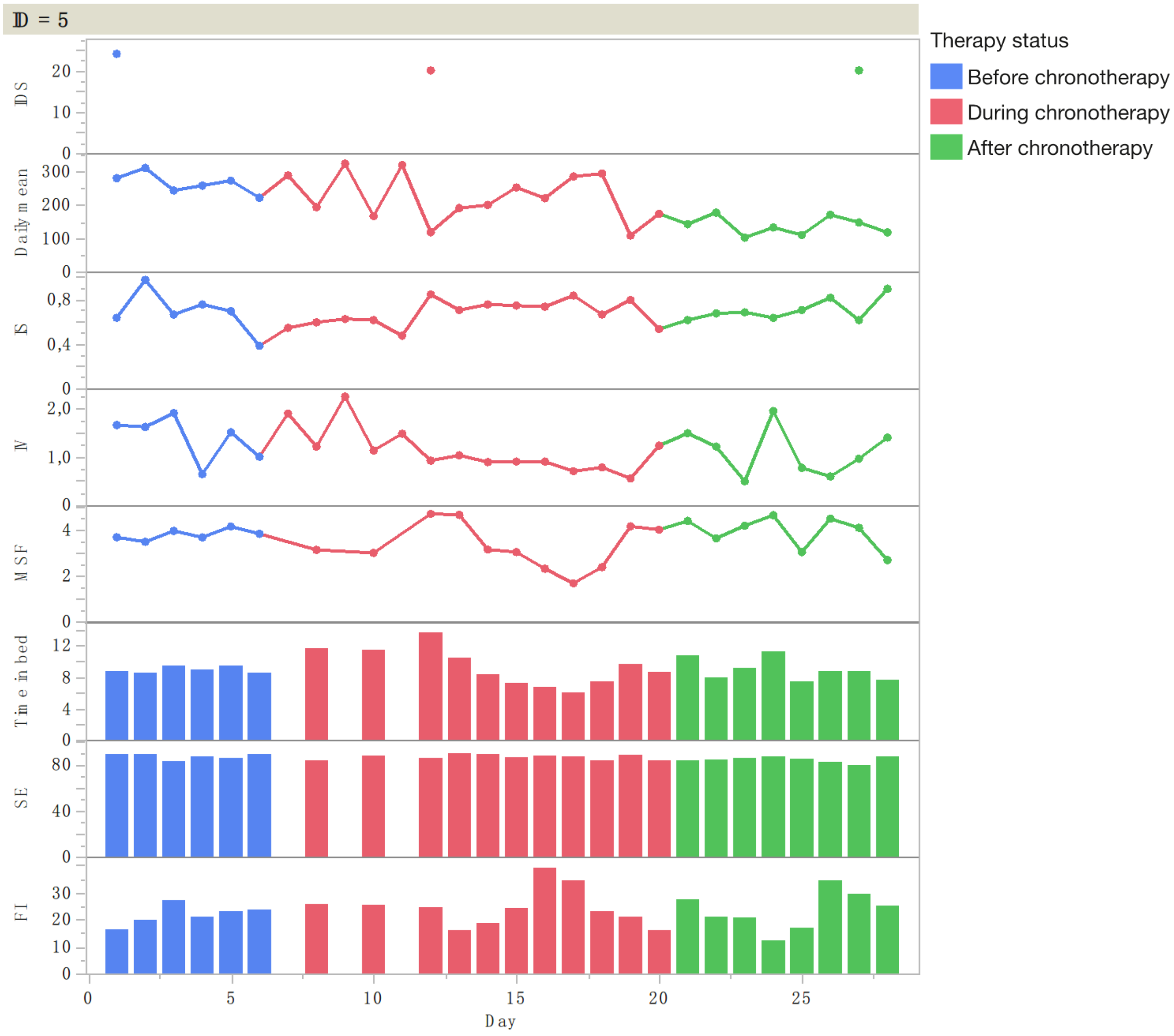
ID = 5
IDS
Daily mean
IS
IV
MSF
Time in bed
SE
FI
Day
Therapy status
Before chronotherapy
During chronotherapy
After chronotherapy

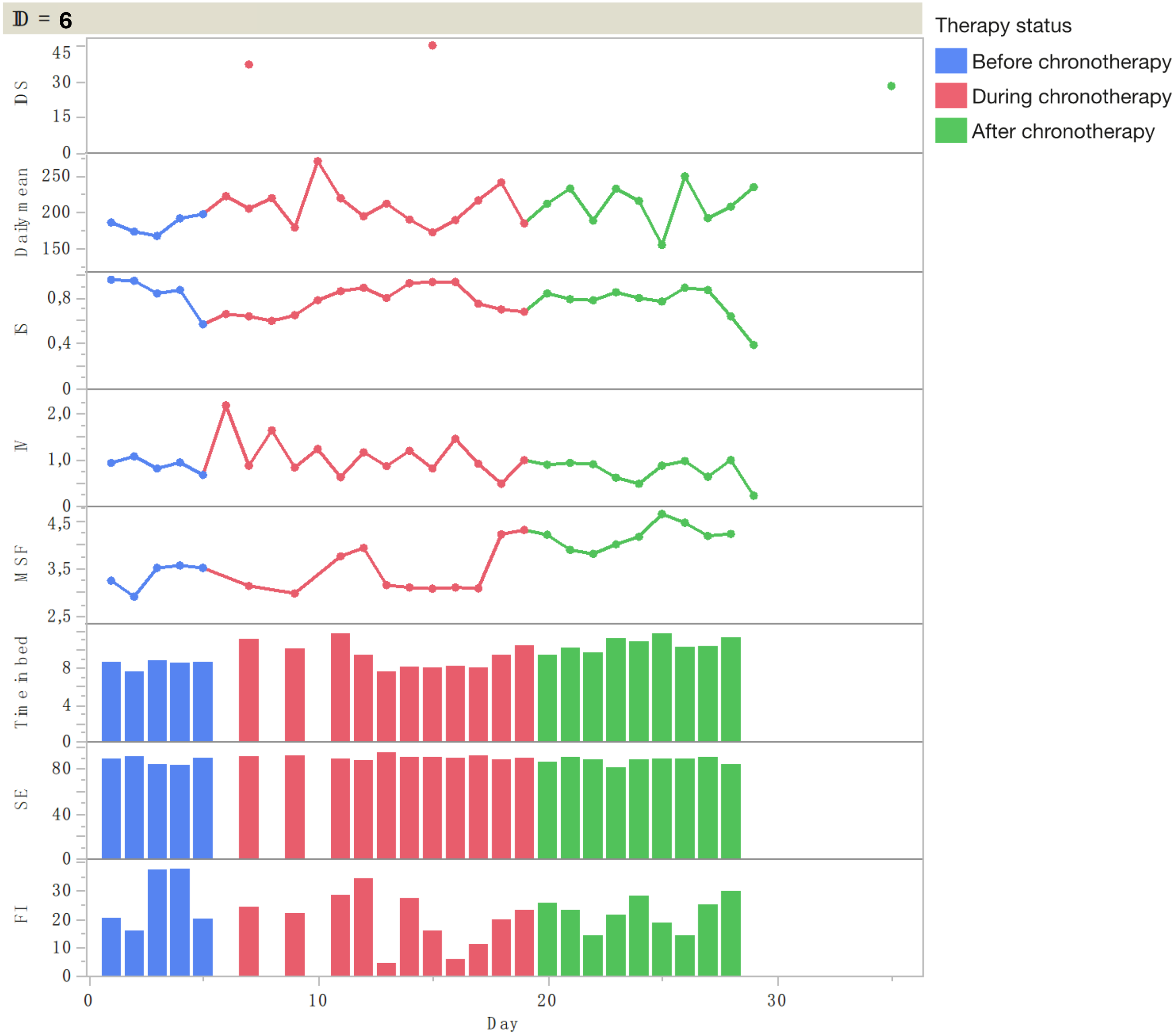
ID = 6
Therapy status
Before chronotherapy
During chronotherapy
After chronotherapy
DS
Daily mean
IS
IV
MSF
Time in bed
SE
FI
Day

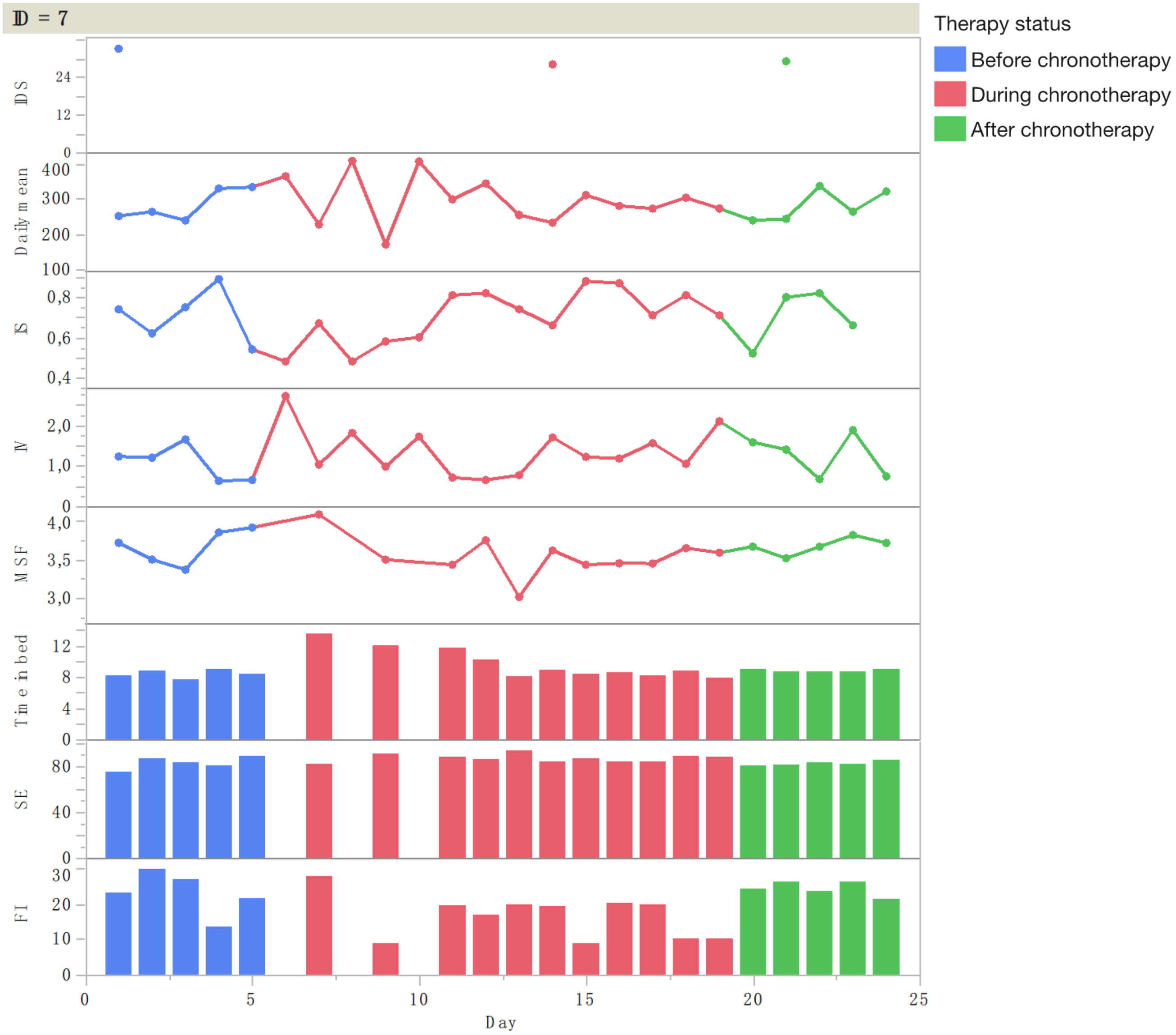

ID = 7
Therapy status
Before chronotherapy
During chronotherapy
After chronotherapy
IDS
Daily mean
IS
IV
MSF
Time in bed
SE
FI
Day

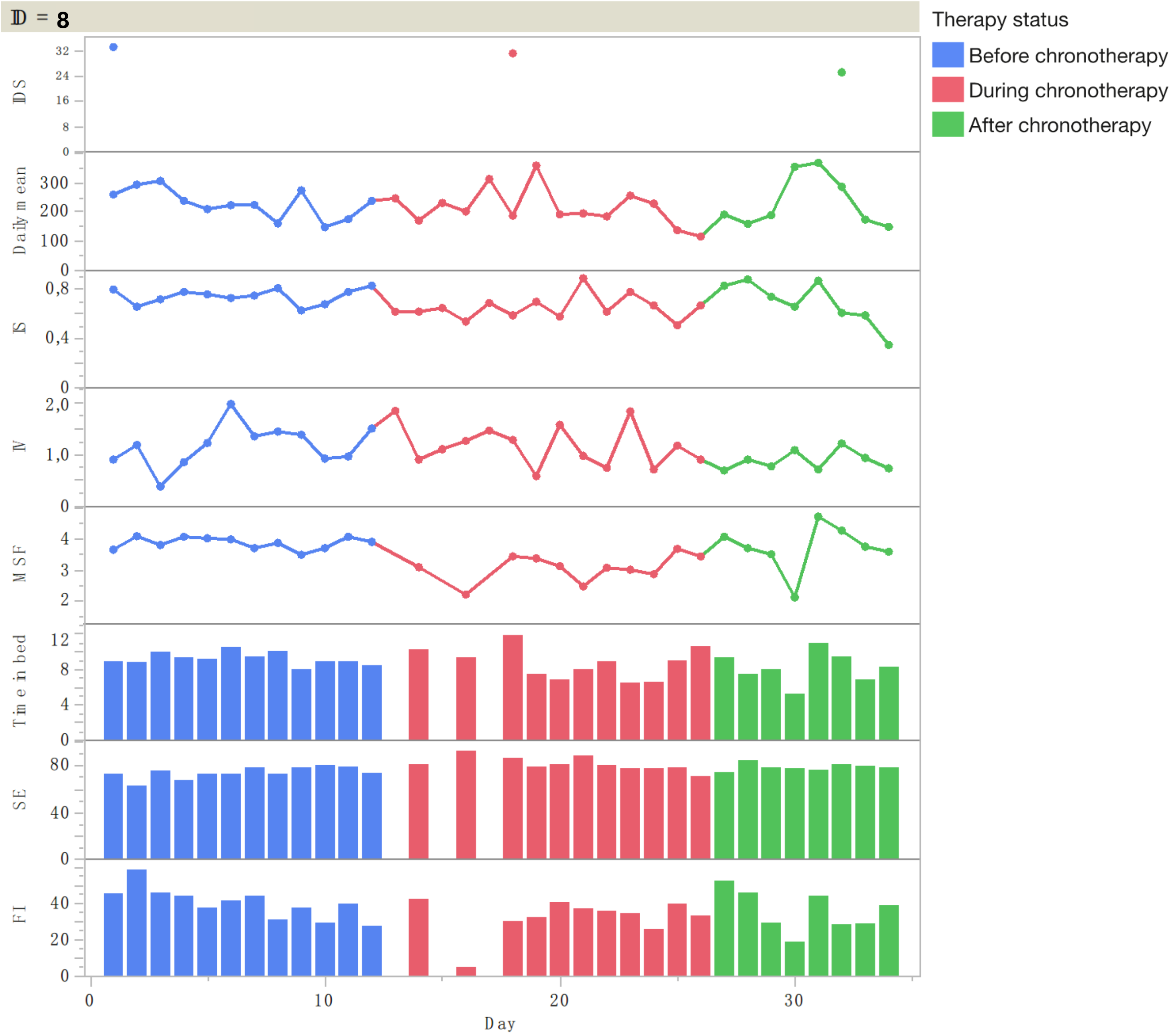

ID = 8
Therapy status
Before chronotherapy
During chronotherapy
After chronotherapy
IDS
Daily mean
IS
IV
MSF
Time in bed
SE
FI
Day

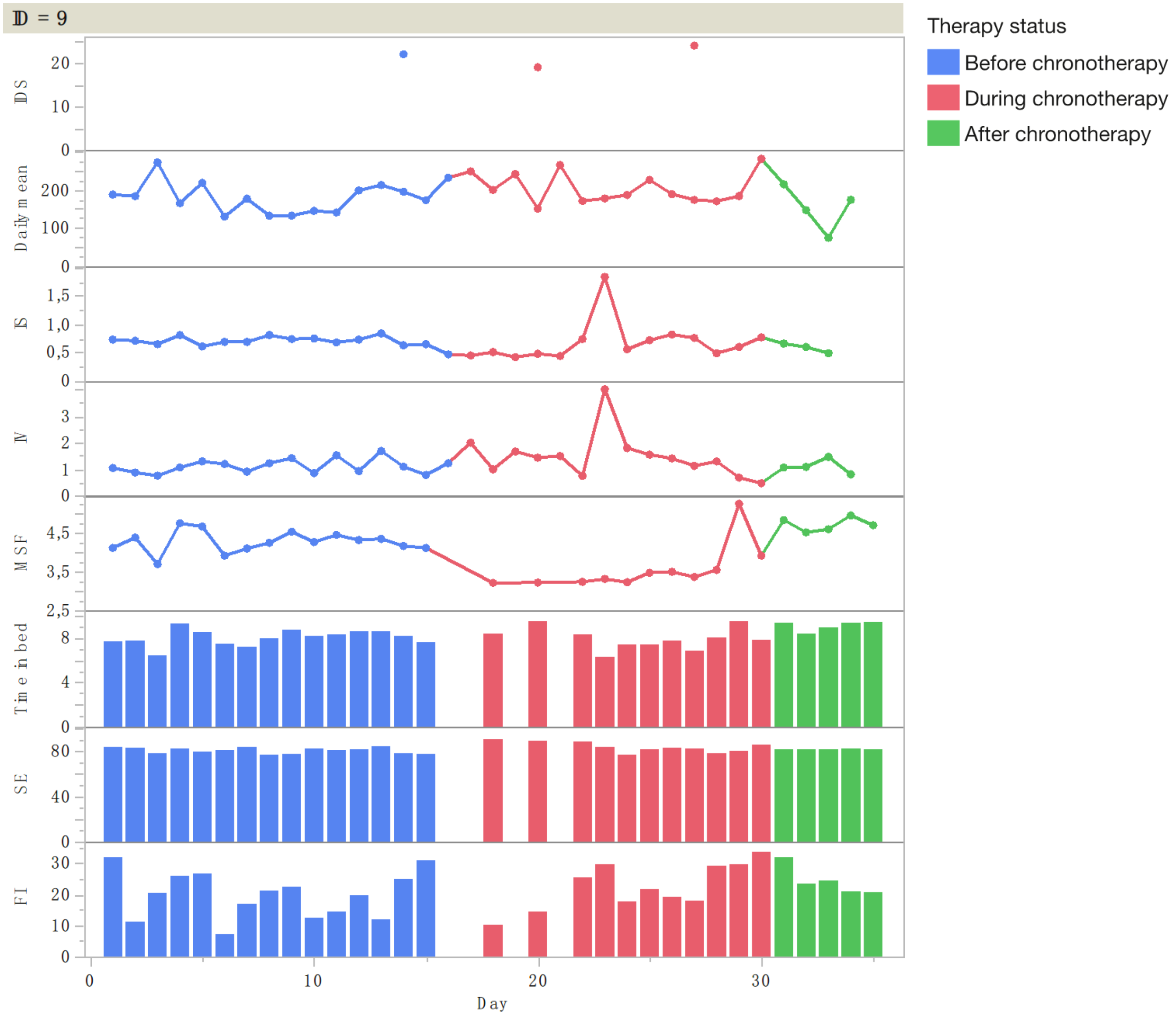

Note: For the IDS, the third assessment on day 27 at the end of chronotherapy was taken as an 'after chronotherapy' measure for the calculation of response/nonresponse.
